# Can pre-procedural MRI signal intensity ratio predict the success of uterine artery embolization in treatment of myomas?

**DOI:** 10.3906/sag-2012-136

**Published:** 2021-06-28

**Authors:** Çağlayan ÇAKIR, Fatih KILINÇ, Muhammed Akif DENİZ, Sema KARAKAŞ

**Affiliations:** 1 Department of Radiology, University of Health Sciences, Bakırkoy Dr. Sadi Konuk Education and Research Hospital, İstanbul Turkey; 2 Department of Internal Medical Sciences, Faculty of Medicine, Dicle University, Diyarbakır Turkey; 3 Department of Obstetrics and Gynecology, University of Health Sciences, Bakırkoy Dr. Sadi Konuk Education and Research Hospital, İstanbul Turkey

**Keywords:** Myoma, embolization, uterine artery, magnetic resonance imaging

## Abstract

**Background/aim:**

Magnetic resonance (MR) images, signal intensity ratios calculated using region of interests (ROI) in T2W images by proportioning the dominant myoma to iliac muscle can aid patient selection and, thus, in achieving better outcomes with the uterine artery embolization (UAE) procedure. The present study investigates the association between the success of UAE treatment with signal intensity (SI) ratio of the dominant myoma to the iliac muscle in MR imaging performed prior to the procedure.

**Materials and methods:**

This is a retrospective study and included 30 patients who admitted to our clinic between February 2017 and July 2019 due to symptoms associated with myoma and who underwent MR imaging before and after UAE treatment. All patients, MR images obtained before UAE treatment and at the 12th month after the procedure were evaluated. In MRI, SI values were calculated by proportioning the dominant myoma to the iliac muscle using circular ROI in T1 weighted (W), T2W, and post-contrast T1W images. In the present study, 50% or more volumetric regression of the myoma with infarction of fibroids (loss of enhancement) at the 12-month follow-up MRI after the procedure was considered a successful procedure.

**Results:**

Myoma volumes calculated in MR images showed significant differences between the MRI performed before UAE procedure and the MRI performed at the 12th month after the procedure (p < 0.0001). SI ratio calculated from pre-procedure T2W MR images was found to be a significant determinant of 50% or more volumetric regression in the myoma after UAE procedure (p = 0.017), T1W, post-contrast T1W images were not statistically significant (p = 0.211).

**Conclusion:**

Our results indicate that SI ratio of the dominant myoma to the iliac muscle calculated using ROI in T2W images of MR studies performed before UAE procedure can predict the success of the procedure.

## 1. Introduction

Uterine leiomyomas are the most common gynecological benign neoplasias of the reproductive age, affecting 20%–30% of women in this period [1]. Leiomyomas, also called uterine fibroids, are generally asymptomatic, although they can cause symptoms like bleeding and anemia in particular as well as frequent urination, abdominal pain, and abdominal swelling.

Uterine artery embolization (UAE), as a minimally invasive method, is used as a treatment because it treats the symptoms, reduces the size of the fibroids and improves the quality of life of patients [1].

Ultrasonography (US) is the first-line imaging modality for uterine myomas, although it often fails to detect lesions smaller than 1 cm in diameter. Transvaginal US has greater sensitivity than transabdominal US in detecting uterine leiomyomas. In consideration of the advantages of magnetic resonance (MR) imaging with regard to detection of myomas, MR can be used to determine number and localization of myomas and even perform volumetric calculations; thus, it is regarded as the best imaging method for uterine leiomyomas.

UAE is a safe treatment option that can be performed by interventional radiologists under angiographic guidance. Signal intensity (SI) ratio of myoma to iliac muscle calculated using circular region of interest (ROI) in addition to routine evaluation of T1W, T2W, and post-contrast T1W images in MRI scans performed prior to UAE can yield important clues about treatment success [2–4].

In this study, our aim was to investigate whether SI ratio, a quantitative parameter obtained by proportioning the myoma to iliac muscle in pre-procedure MRI, can be used as a determinant of 50% or greater regression in myoma volume with infarction of fibroids (loss of enhancement) as an indicator of treatment success in cases undergoing UAE due to myoma.

## 2. Materials and methods

### 2.1. Study design

The study was planned as a single-center retrospective study and included 30 patients who presented to our clinic between February 2017 and July 2019 due to symptoms associated with uterine leiomyoma and who underwent UAE procedure.

Patients who had uterine leiomyoma but did not have complaints associated with myoma such as bleeding, anemia, frequent urination, and abdominal pain and had asymptomatic course, and those who were found to have active pelvic infection or malignancy were not included in the study. All patients provided informed consent for UAE procedure.

For all patients, MR images scanned before UAE procedure and at the 12th month after the procedure, and clinical symptoms experienced before and after the procedure were obtained retrospectively from patient files, computer record systems, and imaging archives.

MRI scans were performed using pelvic superficial coils in a 3T (Tesla) MRI device (Siemens Verio, Malvern, PA, US). Pelvic MR scans included T2W sagittal and axial; T1W sagittal, axial, and coronal; and post-i.v. 0.1 **–** 0.2 mmol/kg Gadobutrol injection T1W sagittal, axial, and coronal sequences. Leiomyoma volumes were calculated based on sagittal and axial images with the formula: height x width x length x π/6. In those cases having multiple myomas, total myoma volume was calculated by adding the volume of each myoma.

**Figure 1:**
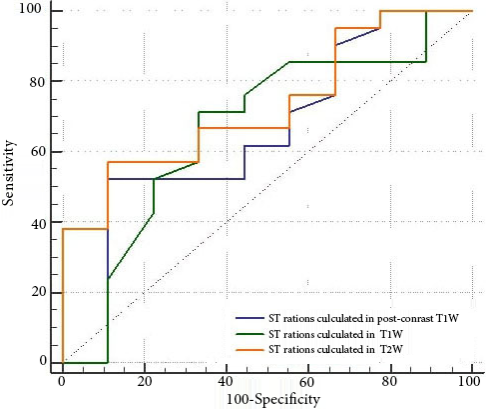
ROC curves of SI ratios calculated in T1W, T2W, and post-contrast T1W images for prediction of 50% or greater volumetric regression following UAE, i.e. successful embolization procedure.

**Figure 2:**
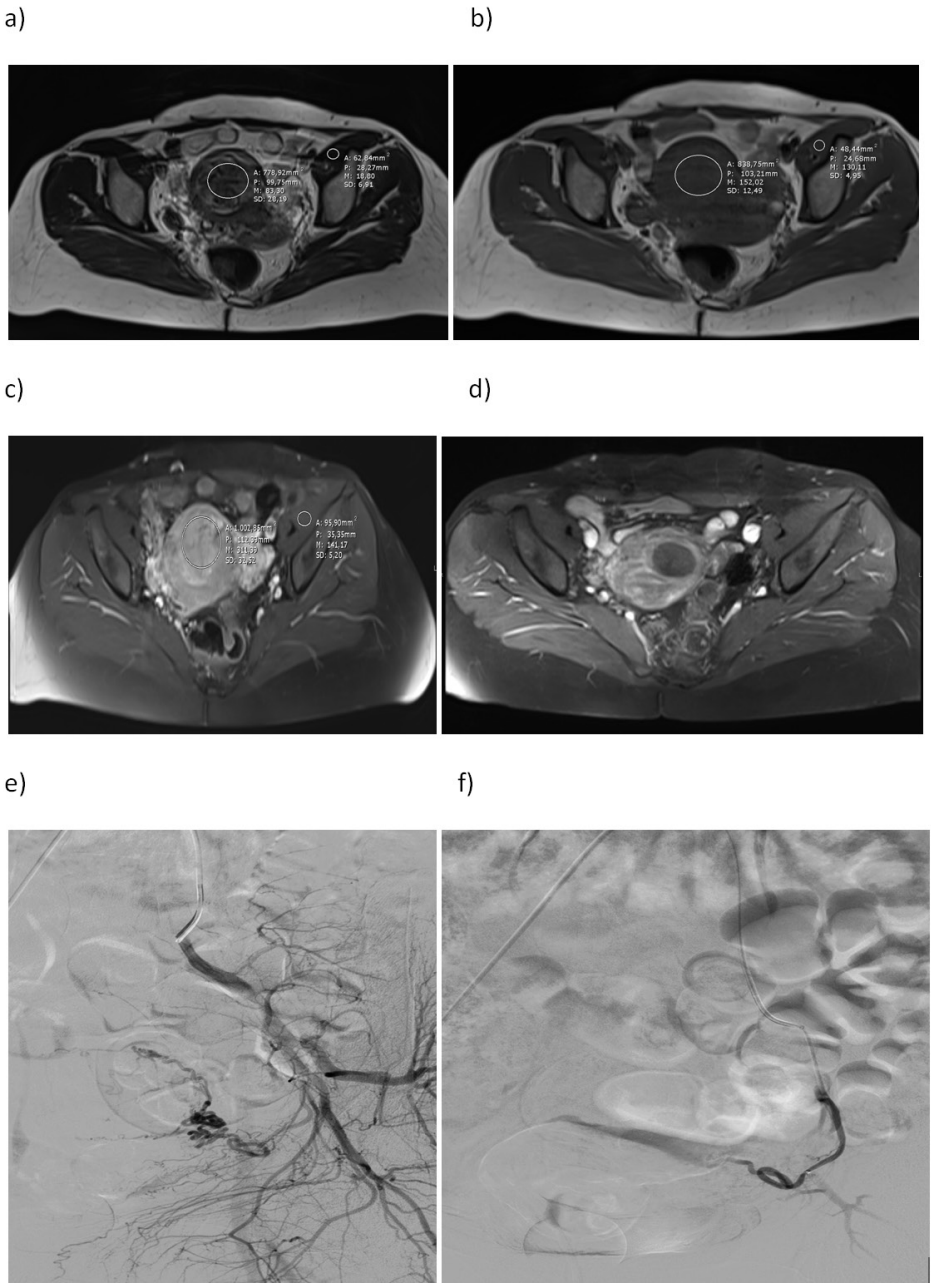
In the example case, SI ratios calculated before the embolization procedure were 4.61 in T2W, 1.16 in T1W, and 2.1 in post-contrast T1W MR images. Myoma volume was 45 cc before UAE and regressed to 10 cc after the procedure (ad). In addition, Figures e-f show DSA images obtained selectively from the left uterine artery before and after embolization.

In pre-UAE T1W and T2W MR images, ROIs were placed so as not to include necrotic and cystic areas of myomas, and a mean SI value was obtained (Figure 1). Similarly, mean SI values were obtained from the center of right or left iliac muscle using ROIs. SI values calculated in T1W, T2W, and post-contrast T1W MR images were proportioned between dominant myoma and iliac muscle to obtain the SI ratio (Figure 2 a-f). A successful procedure was accepted as presence of 50% or greater volumetric regression of the myoma and infarction of fibroids (loss of enhancement) at the 12-month follow-up MRI. Two radiologists who had at least 5 years of experience performed all measurements. 

For the embolization procedure, all cases received sedative anesthesia as perioperative analgesia. UAE procedures were performed under fluoroscopic (Allura FD 20/20, Philips Medical System, Best, Netherlands) guidance. For the procedure, a 5 French (Fr) arterial sheath was placed in the right common femoral artery under ultrasonographic guidance. Then, internal iliac arteries were selectively catheterized using Cobra diagnostic catheter (5 Fr) [Cordis, Johnson and Johnson, USA] for the left uterine artery and Simmon 1 diagnostic catheter (5 Fr) [Cordis, Johnson and Johnson, USA] for the right uterine artery. Distal embolization was achieved using microsphere agents (Embosphere Microspheres; Merit Medical, USA) with sizes varying between 500 **–** 700 microns and 700 **–** 900 microns and with super selective catheterization of ascending branch of the uterine artery by inserting 2.8 Fr Rebar 27 (Medtronic, Irvine, CL, USA) or 2.8 Fr EmboCath Plus (Biosphere Medical, France) microcatheter through the existing diagnostic catheter in ipsilateral oblique projection. In control angiograms after embolization, and this was accepted as complete embolization and end point (complete stasis, sluggish flow, or pruned tree appearance). 

In one patient with uterine-ovarian artery anastomosis, the procedure was not satisfied by performing coil embolization from the proximal of the ovarian artery. None of the patients developed any complications associated with the endovascular procedure.

Foley catheter was removed following the procedure, and patients received i.v. analgesics until the 6th post-op hour. After the procedure, total of 29 patients were discharged one day after bed rest and one patient could be discharged one week later due to urosepsis. All patients received ciprofloxacin 500 mg peroral twice daily and nonsteroid antiinflammatory medication and proton pump inhibitors for 10 days. 

### 2.2. Statistical analysis

Categorical variables are expressed as frequency and percentage values. Continuous variables are expressed as mean, standard deviation, median, and minimum and maximum values. Normality assessment of continuous variables was made with Kolmogorov–Smirnov test. Nonnormally distributed dependent variables were analyzed with Wilcoxon signed rank test, and associations between the variables were analyzed with Spearman’s correlation test. ROC analysis was employed to determine the SI cut-off values in T1W, T2W, and post-contrast T1W MR images for prediction of 50% or more volumetric regression in myoma after UAE. Comparison of areas under ROC curves was made using the method described by De Long et al. All analyses were performed with MedCalc Statistical Software version 18 (MedCalc Software bvba, Ostend, Belgium). 

## 3. Results

In the present study, a total of 30 patients underwent UAE procedure due to symptoms associated with uterine fibroids. Patient ages ranged between 23–50 years (mean: 41.23).

Patients’ clinical symptoms were uterine bleeding with frequent and irregular intervals (menometrorrhagia) (88%) and symptoms associated with compression (12%).

Dominant myomas had intramural (88%), submucosal (8%), and subserosal (4%) localizations. The largest myoma in our series had a volume of 1020 cc, and the smallest one had a volume of 17 cc. At the 12th month follow-up MRI after the procedure, the largest myoma volume was 810 cc and the smallest myoma volume was 2 cc. There was a statistically significant difference between the measured myoma volumes in MRI before UAE procedure and the volumes measured at post-procedure 12th month MRI (p < 0.0001). Examination of mean and median values before and after UAE procedure showed that the procedure resulted in reduced volume of myoma (Table 1).

**Table 1:** Myoma volume before and after UAE procedure.

	(n)Mean ± SDMedian (Min-max)	p
Myoma volume before (MR)	(n = 30)241.7 ± 264.07179–(17–1020)	p < 0.0001
After UAE procedure (MR)	(n = 30)120.5±173.3865.5–(2–810)	

Wilcoxon signed rank test.

ROC analysis was employed to determine cut-off SI ratios in T1W, T2W, and post-contrast T1W MR images for prediction of 50% or greater volumetric reduction in myoma following UAE procedure. No statistically significant cut-off SI value in T1W (p = 0.174) or post-contrast T1W (p = 0.211) images could predict 50% or greater volumetric reduction of myoma following UAE procedure. On the other hand, AUC for SI ratio in T2W images was calculated as 0.730, and SI ratio in T2W images was found as a significant determinant of 50% or greater volumetric reduction following UAE (p = 0.017) (Figure). SI cut-off value in T2W images was calculated as 2.8. Using this cut-off value, 57.1% of cases with 50% or greater volumetric reduction, and 88.9% of cases with less than 50% volumetric reduction could be accurately predicted. Overall accuracy rate (diagnostic effectiveness) for this cut-off value was 66.67%.

A review of successful and unsuccessful cases after UAE, i.e. those with 50% or greater volumetric reduction and those with less than 50% volumetric reduction, respectively, showed that of all the cases for whom successful treatment is predicted according to the cut-off value, 92.3% actually had treatment success, whereas of all the cases for whom treatment failure is predicted, 47.1% actually had treatment success. These findings are summarized in Table 2 and Table 3. 

**Table 2:** Descriptive statistics.

	(n)Mean ± SDMedian (Min-max.)
Age	(n = 30)41.23 ± 4.9242–(23–50)
Myoma volume before (MR)	(n = 30)241.7 ± 264.07179–(17–1020)
After UAE Procedure (MR)	(n = 30)120.5 ± 173.3865.5–(2–810)
SI ratios calculated in T2W	(n = 30)3.2 ± 1.612.67–(1.04 –8)
SI ratios calculated in T1W	(n = 30)1.11 ± 0.241.1–(0.65–2)
SI ratios calculated inpost-contrast T1W	(n = 30)2 ± 0.581.96–(0.48–3.6)

**Table 3:** Prediction of 50% or greater volumetric reduction in myomas using SI ratios calculated in T1W, T2W, and post-contrast T1W MR images.

Success	AUC%95CI	p	Cut-off%95CI	Sensitivity %95CI	Specificity %95CI	PPV%95CI	NPV%95CI
SI ratios calculated inpost-contrast T1W	0.651(0.45–0.815)	0.211	>2(>1.5–>2.86)	52,4(29.8–74.3)	88.9(51.8–99.7)	91.7(62.4–98.6)	44.4(32.6–57.0)
SI ratios calculated in T1W	0.664(0.46–0.825)	0.174	>1.04(>0.9–>1.4)	71.4(47.8–88.7)	66.7(29.9–92.5)	83.3(65.6–92.9)	50.00(30.6–69.4)
SI ratios calculated in T2W	0.730(0.53–0.875)	0.017	>2.8(>2.2–>3.2)	57.1(34.0–78.2)	88.9(51.8–99.7)	92.3(64.6–98.8)	47.1(34.0–60.5)

ROC analysis.

## 4. Discussion

The purpose of the UAE procedure is selective catheterization of the uterine artery and embolization of the arterioles that supply the myoma by using appropriately sized ideal embolic agents, therefore cutting off the blood supply to central and peripheral parts of the myoma. Currently, UAE can yield successful results in appropriately selected cases with symptomatic myomas [5].

The most important indicator of clinical improvement following UAE is regression of the volume of the myoma and infarction of fibroids (loss of enhancement). A review of the related literature shows varying volumetric reduction rates between 42%–83% following UAE. This rate was 50% on average in our series, which is consistent with the literature [5,6]. Previous studies reported reduction in uterus size by 43%–58% after the procedure [7–9]. This rate was 51% in our series.

Since MRI can excellently document zonal anatomy of the uterus [10], we used MRI for evaluation of our patients both before and at the 12th month after the UAE procedure in the present study. Myomas appear as well-demarcated lesions in MR images. High-resolution T2W images without fat suppression obtained on three planes (axial, coronal, and sagittal) are the most reliable sequences for detecting myomas [11,12].

T2W-hyperintense myomas are comprised of compact smooth muscle cells and do not contain collagen. Additionally, they show marked contrast uptake due to increased vascularity and intense cellularity, and therefore have the best response to UAE treatment [13–16]. Several studies have documented that T2-hyperintense myomas show greater volumetric reduction after UAE treatment when compared to isointense or hypointense myomas [17–19]. However, it should be kept in mind that myomas with cystic degeneration do not show contrast uptake despite having high signal intensity in T2W images. Myomas with myxoid degeneration, on the other hand, show quite high T2W signal intensity and minimum contrast uptake [20,21]. Degenerated myomas often have irregular borders and show various signal intensities in T2W images depending on their content, such as low signal intensity in the presence of hyaline and calcified areas.

Calculation of signal intensity ratio in MR images is a practical method in daily routine. Proportioning the dominant myoma to the right or left iliac muscle is a quantitative evaluation method with potential utility [22]. Proportioning of the dominant myoma to iliac muscle in T2W images in particular is an important tool for prediction of successful embolization, which means 50% or greater volumetric reduction of the myoma (Table 4). In our study, we calculated the SI ratio cut-off value in T2W images as 2.8. Any higher value can be associated with successful embolization. Sensitivity and specificity of SI ratio in T2W images at this cut-off value were 57.1% and 88.9%, respectively, and these rates are quite satisfactory. In addition, using this SI ratio cut-off value in T2W images, treatment outcome could be accurately predicted in 66.7% of cases.

**Table 4:** Association of SI ratio in T2W images with volumetric change in myomas before and after UAE procedure.

		SI ratio in T2W images
Volumetric change inmyomas reduction rate	Rho	0.470**
	P	0.009
	N	30

Spearman’s correlation analysis:

In T1W images, myomas mostly appear isointense with myometrium and iliac muscles. A very small portion of myomas show hyperintense signal in T1W images, and this feature indicates a lack of significant benefit from UAE treatment [8].

Myomas can show varying rates of contrast uptake in post-contrast T1W images. Some studies have reported that this feature is a significant determinant of volume regression [22].

In our series, SI ratios calculated by using ROIs in T1W and post-contrast T1W MR images were not found to be statistically significant determinants of 50% or greater volumetric reduction following UAE. This might be related to difference in delay times and different scan times in devices.

Apart from the number and signal intensity of myomas, intracavitary, broad-ligament, and cervical localizations in MRI are indicators that the myoma is not appropriate for UAE treatment; patients with myomas localized to these areas were excluded from the present study. Localization of the myoma is an important factor; myomas with submucosal localization have been reported to benefit more from UAE treatment [23]. In our series, 88% of the myomas had intramural localization, and 90% of such myomas achieved 50% or greater volumetric regression after UAE.

In addition to our todays knowledge, UAE with quantitative perfusion and diffusion MR imaging to monitor early treatment response is feasible and might contribute to providing confidence for the patients in treatment success [24,25].

There are several limitations to our study. One was that there might have been differences in determination of the circular ROIs, leading to differences in the calculation of SI ratios. Additionally, the study included a small group of patients. Signal ratios of dominant myomas and volumetric regression in the total myoma were taken into account. More detailed results can be obtained with greater sample size and multiple analyses for each myoma. 

In conclusion, when used in conjunction with the number, localization, and morphology of myomas in pelvic MR images, signal intensity ratios calculated using ROIs in T2W images by proportioning the dominant myoma to iliac muscle can aid patient selection and, thus, in achieving better outcomes with the UAE procedure.

## Funding

We wish to confirm that there are no known conflicts of interest associated with this publication and there has been no significant financial support and funding for this work that could have influenced its outcome. 

## Availability of data and materials

All data and materials were used in our original article and the statistical analysis was done completely. The data and materials mentioned in the original article are completely real. All procedures performed in the studies involving human participants were in accordance with the ethical standards of the institutional and/or national research committee and with the 1964 Helsinki Declaration and its later amendments or comparable ethical standards. This study was approved by the ethics committee of our university.

## Informed consent

Additional informed consent was obtained from all individual participants for whom identifying information is included in this article.
